# The regulatory networks of the Hippo signaling pathway in cancer development

**DOI:** 10.7150/jca.62402

**Published:** 2021-08-28

**Authors:** Maonan Wang, Manli Dai, Dan Wang, Wei Xiong, Zhaoyang Zeng, Can Guo

**Affiliations:** 1 NHC Key Laboratory of Carcinogenesis, Hunan Cancer Hospital and the Affiliated Cancer Hospital of Xiangya School of Medicine, Central South University, Changsha, Hunan, China.; 2 Key Laboratory of Carcinogenesis and Cancer Invasion of the Chinese Ministry of Education, Cancer Research Institute, Central South University, Changsha, Hunan, China.; 3 Hunan Food and Drug Vocational College, Changsha 410036, China.

**Keywords:** Hippo signaling pathway, YAP, Tumor development

## Abstract

The Hippo signaling pathway is a relatively young tumor-related signaling pathway. Although it was discovered lately, research on it developed rapidly. The Hippo signaling pathway is closely relevant to the occurrence and development of tumors and the maintenance of organ size and other biological processes. This manuscript focuses on YAP, the core molecule of the Hippo signaling pathway, and discussion the upstream and downstream regulatory networks of the Hippo signaling pathway during tumorigenesis and development. It also summarizes the relevant drugs involved in this signaling pathway, which may be helpful to the development of targeted drugs for cancer therapy.

## Introduction

In addition to the classical eight cancer-related signaling pathways, with the development of scientific research, more and more signaling pathways have been found to play a significant role in the development of cancer 1. The Hippo pathway found in Drosophila is a crucial entry point for understanding the molecular mechanisms that control organ growth during development and regeneration 2-4. The intracellular environment's inhibitory growth signal activates the Hippo signaling pathway, causing a series of Kinase cascade phosphorylation. MST1/2 (mammalian STE20-like protein kinase) phosphorylation and cofactor SAV1 (human Salvadorhomology 1) promote LATS1/2 (large tumor suppressor1/2) phosphorylation and cofactor MOB1 (MOB kinase activator) of downstream 5-7. Then, the phosphorylation of downstream effector YAP (Yes-associated protein) /TAZ (Tafazzin) could be increased. Phosphorylated YAP/TAZ could be in the cytoplasm through binding 14-3-3 proteins 8,9 and it could promote proteasome degradation, eventually inhibiting cell proliferation 7,10,11. However, when the cells become cancerous, Hippo pathways are suppressed. Once the “switch” closes, the unphosphorylated YAP/TAZ may enter the nucleus, as a co-transcriptional coactivator 12. Then, it binds to the TEA domain family members (TEAD) transcription family and promotes the expression of oncogenes associated with tumorigenesis. Finding the switching mechanism related to the Hippo pathway is very necessary for the fight against cancer 13,14. This review focuses on the upstream and downstream regulatory networks of Hippo pathways, biological effects in tumor formation, and advances in therapeutic cancer targets in pathways.

## The role of Hippo signal pathway

The Hippo signaling pathway was first discovered in Drosophila and had a high evolutionary conservation 15. In the early stages of cancer, a large number of cancer-derived missense mutations occurred in the highly conserved Hippo pathway, and these mutations caused the Hippo pathway to be inhibited 16. The kernel components of the Hippo pathway include the upstream kinase MST1/2 and LATS1/2, cofactor SAV1, spherical scaffold protein MOB1A/B 17, transcription coactivator YAP and TAZ, and transcription enhancing association domain TEADs. SAV1 is the primary molecule in the Salvador pathway, LATS1/2 is the central molecule in the Warts pathway, and MST1/2 is the central molecule in the Hippo pathway. Therefore, the Hippo signaling pathway can also be recorded as the Salvador/Warts/Hippo pathway.

**Figure 1:**
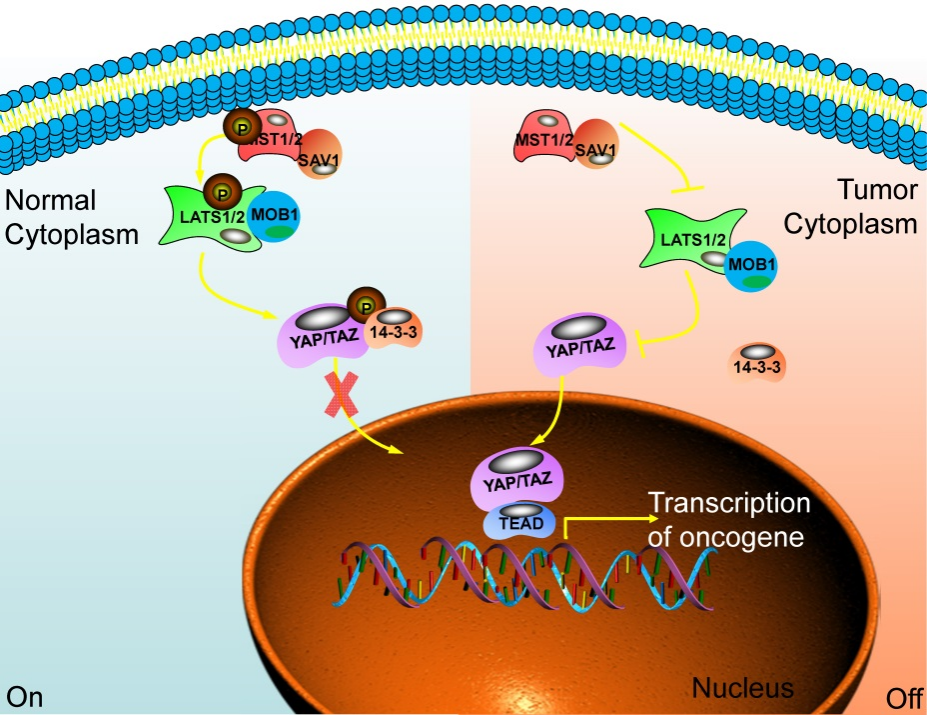
** The main component of the Hippo signaling pathway.** Current research shows that there are six key molecules in the Hippo signaling pathway, namely MST1/2, SAV1, LAST1/2, MOB1, YAP and TAZ. In normal cells, the Hippo pathway is activated, and YAP/TAZ is retained in the cytoplasm through some cascade reactions. In tumor cells, this pathway is closed and YAP/TAZ enters the nucleus to play the role of transcriptional regulators and promote the transcription of multiple oncogenes. Ultimately, promote the occurrence of tumors.

When the Hippo pathway is activated, the upstream signal molecule activates MST1/2 and binds to the co-regulatory protein SAV1. Then, phosphorylated LATS1/2 binds to the co-regulatory protein MOB1. After that, the phosphorylation of YAP/TAZ has been activated and binds to the scaffold protein 14-3-3. The YAP/TAZ will be in the cytoplasm and is degraded by the proteasome after ubiquitination. Finally, the biological function of YAP/TAZ is inhibited 18. When cancer happens, the Hippo pathway is blocked or inactivated, and the phosphorylation cascade is affected. The unphosphorylated YAP/TAZ enters the nucleus from the cytoplasm. It combines with the TEADs transcription family, acting as a transcriptional coactivator, synergistically promoting the expression of target genes, and enhancing cell proliferation and anti-apoptosis 19,20 (Fig. 1).

It is worth noting that the change of MST1/2 does not necessarily cause changes in the Hippo pathway. It could also regulate proteins that do not participate in the Hippo pathway, such as NDR1/2 (Late embryogenesis abundant (LEA) hydroxyproline-rich glycoprotein family), forkhead box O1 (FOXO1)), etc. Similarly, activation of YAP/TAZ does not necessarily require MST1/2. It can also act on LATS1/2 or be directly regulated by other proteins. For example, Hppy and Wsn could inhibit the transcriptional activity of Yki by activating Wts kinase activity. Not all proteins and functions regulated by MST1/2 are therefore definable as Hippo pathways, specifically regulating YAP or LATS1/2 kinase activity only. The protein pathway of /TAZ transcriptional activity is classified as the Hippo signaling pathway 21.

## The upstream regulatory networks of the Hippo signaling pathway

**Figure 2:**
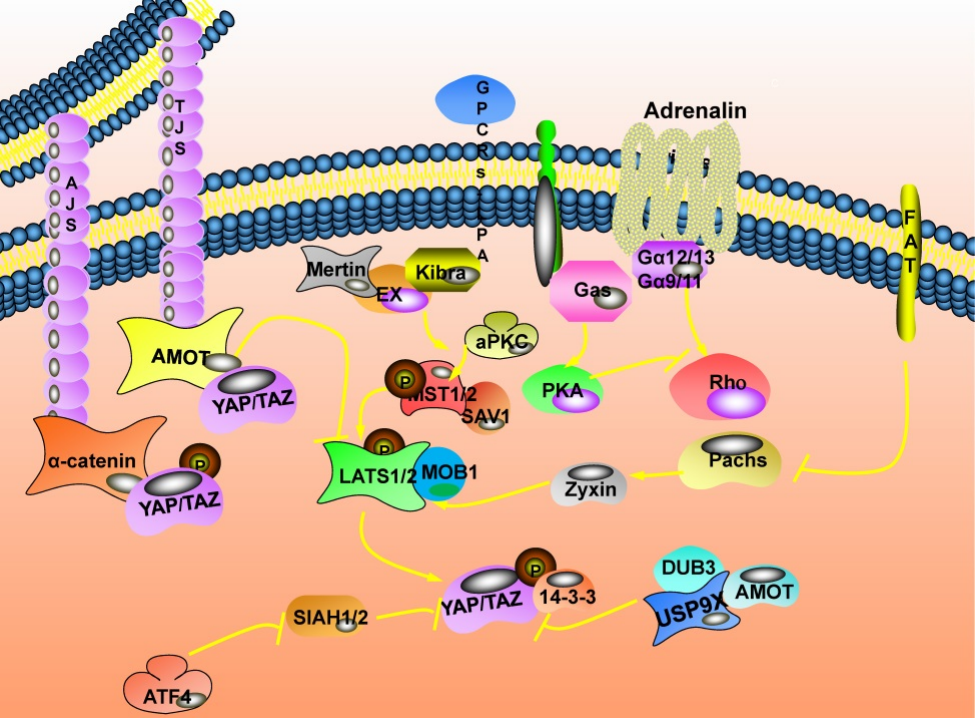
** The upstream regulatory network of the Hippo signaling pathway.** Cell junction related proteins are located on the cell membrane. Mainly divided into tight junctions (TJs) and adhesive connections (AJs). Ubiquitin specific peptidase 9 X-linked (USP9X) in the ubiquitin-specific protease family (DUBs) could bind to and deubiquitinate angiomotin (AMOT). FAT atypical cadherin 1 (FAT) is the first transmembrane receptor identified to affect the expression of the Hippo pathway, and it is also a transmembrane apex determinant. The mechanism by which FAT affects the expression of the Hippo pathway depends on the relationship between FAT and Expande (Ex).

The Hippo signaling pathway is very evolutionarily conserved and is involved in maintaining tissue homeostasis. When the pathway is closed by extracellular signals, it often induces cancer. Abnormal expression of the Hippo pathway has been detected in various cancers, accompanied by changes in extracellular signals. With the deepening of research, more and more upstream regulatory networks of the Hippo signaling pathway have been unearthed 22 (Fig. 2).

### Regulation of cell connection on the Hippo signaling pathway

There are direct or indirect connections between cells in multicellular organisms, and cell connection is an important area where cells are connected. Cell junction related proteins are located on the cell membrane. Mainly divided into tight junctions (TJs) 23 and adhesive connections (AJs) 24. Angiomotin (AMOT) is complicated in the regulation of tight junctions and tubular formation, migration of vascular endothelial cells, and signal transduction of the Hippo pathway. There are two molecular mechanisms for AMOT to regulate YAP/TAZ activity: one is that AMOT directly binds YAP/TAZ and retains them in the cytoplasm to inhibit its activity expression. This interaction does not rely on Hippo pathway-mediated YAP/TAZ phosphorylation 25. The other is the upstream kinase that could act on the Hippo pathway. AMOT could directly bind to LATS2 and up-regulate the expression of LATS2, ultimately inhibiting the expression activity of YAP/TAZ 26; it could also stimulate the activation of MST1/2. Then, the phosphorylation of LATS1/2 is promoted through the cascade phosphorylation reaction of the Hippo pathway to activate LATS1/2, and finally, the expression activity of YAP/TAZ is inhibited 27. Besides, the changes in the ubiquitin chain topology caused by ubiquitination could change protein stability, membrane transport, and cellular localization. USP9X in the DUBs (ubiquitin-specific protease family) could bind to and deubiquitinate AMOT. Finally, inhibit the expression activity of YAP/TAZ 28. Transmembrane proteins (Crumbs, Crb) participate in regulating the polarity of the top-bottom layer of the cell. They could bind to Expande (Ex) through the FBM region to change its subcellular location and stability, thereby regulating the expression of swimming genes 29. Merlin (Mer) could form a complex with Ex and enriched in the top layer of epithelial cells. It could cooperate with Kibra to bind SAV1 and participate in the signal transduction of the Hippo pathway 7,30. Also, the aPKC complex formed by aPKC, Bazooka, and Par6 could antagonize Scrib to promote the activation and expression of YAP/Hippo 31.

### Regulation of Planar Cell Polarity on the Hippo signaling pathway

The three-dimensional morphological distribution of cells in tissues usually shows axiality, and the distribution is not random or uniform. When cells' subcellular structure is distributed asymmetrically along one or several axes, this phenomenon is called cell Polarity 32^.^ Planar Cell Polarity (PCP) also affects the expression of the Hippo pathway 33,34. Atypical Cadherin plays an essential role in the process of biological development and tissue morphogenesis. FAT is the first transmembrane receptor identified to affect the expression of the Hippo pathway, and it is also a transmembrane apex determinant 35.

Here, the mechanism by which FAT affects the expression of the Hippo pathway depends on the relationship between FAT and Ex: When the FAT, Ex and Hippo pathways are regulated in a linear cascade, Fat mediates the Hippo pathway by affecting the subcellular localization and stability expression of Ex 36; when FAT and Ex are in parallel and coordinated regulation, it directly promotes the phosphorylation of LATS1/2 to mediate the expression of the Hippo pathway 37. Besides, FAT could also change the stability of LATS1/2 through Dachs and Zyxin to mediate the expression of the Hippo pathway 38.

### Regulation of cell membrane receptors on the Hippo signaling pathway

The receptors distributed on the cell membrane will activate or inhibit the relevant signal pathways when they feel external stimulus signals and affect cell proliferation or apoptosis 39. G protein-coupled receptors and insulin receptors are essential cell membrane receptors that mediate extracellular signal transmission into the cell 40. The Hippo pathway is regulated by upstream membrane protein receptors and could respond to extracellular growth signals 41. The serum proteins in the serum could bind to their G Protein-Coupled Receptors (GPCRs) on the membrane and activate the expression of YAP/TAZ through Rho GTPase 42. Thrombin could promote the conversion of soluble fibrinogen into insoluble fibrin. It could also activate their G protein-coupled receptor-protease activated receptor (PARs) on the cell membrane and activate the expression of YAP/TAZ 43. The crosstalk mechanism between insulin and insulin like growth factor 1 (IGF-1) signaling pathway in pancreatic cancer inhibits the expression of phosphatidylinositol 3-kinase (PI3K) explicitly, effectively reducing YAP/TAZ 44. In addition, vascular endothelial growth factor (VEGF) could activate VEGF receptors and inhibit the phosphorylation of LATS1/2 and YAP 35.

### Regulation of the cellular mechanical environment on the Hippo signaling pathway

The cellular mechanical environment includes various physical forces that may impact the cell and other surrounding environments. This may affect the internal or external forces generated by the cell. Depending on the composition of the tissue and surrounding matrix, the hardness of the matrix, the size of the available space, as well as the considerable changes in the cell microenvironment throughout the body, the cellular mechanical environment play a significant part in the process of cell proliferation, survival and differentiation, tissue regeneration and wound repair 22. Abnormal mechanical transduction may lead to the activation of key signaling pathways and trigger abnormal cell behavior, potentially affecting the development of chronic diseases 45. The downstream effector YAP/TAZ in the Hippo signaling pathway not only responds to mechanical stimuli (such as tissue stretching, cell density and area, cell adhesion and extension, cell contact, and extracellular matrix hardness, etc.) but also an essential regulator of cell response to these stimuli 46.YAP/TAZ translates a series of signals from splicing pressure to cell morphology and extracellular matrix hardness and converts them into cell-specific transcriptional programs. This mechanism of transduction is essential to drive stem cell behavior and regeneration 47.

It is worth noting that YAP/TAZ's various mechanical signal regulators are mostly concentrated in the actin cytoskeleton. Actomyosin is the main component of cells that respond to mechanical stimuli. The joint action of the nuclear skeleton linker and cytokine mediates mechanical force transfer to the nucleus through actin cells 48. When cells grow at high density, the connections between cells increase, and the upstream kinase LATS1/2 of the Hippo signaling pathway is activated, causing YAP/TAZ phosphorylation and failing to enter the nucleus. In addition, when the cells growing on the soft substrate do not have enough area to stretch, the mesenchymal stem cells will differentiate into adipocytes. The cell morphology becomes round, and YAP/TAZ is mainly distributed in the cytoplasm; on the contrary, mesenchymal stem cells differentiate into osteoblasts, and the cell morphology is flat, YAP/TAZ mainly located in the nucleus 47,49.

However, the interaction between the Hippo pathway and cell tension still needs further exploration. Sun et al. proposed a YAP/TAZ mechanical sensing calculation model, exploring the multi-scale relationship between the adhesion signal and the expression abundance of YAP/TAZ. This model converts the mechanical properties of the extracellular matrix into biochemical signals through adhesion and cascades intracellular signals related to cytoskeleton dynamics to perform molecular level disturbance and sensitivity analysis. It provides an analytical platform for looking at YAP/TAZ activity carefully in the context of combining varying signal pathways 50.

### Regulation of oxidative stress on the Hippo signaling pathway

Oxidative stress is the state of imbalance between oxidation and antioxidant when cells need antioxidants to neutralize excessive free radicals 51. Protein kinase AMP-activated catalytic subunit alpha 1 (AMPK) is a crucial regulator of bioenergy metabolism, and the increase of adenine phosphoribosyl transferase (AMP)/ATP content in the body could activate AMPK 52. Under hypoxic conditions, the content of ATP decreases, and the cells are in an energy-deficient state. There are two molecular mechanisms for activated AMPK to regulate the activity of YAP. One is that AMPK activates and directly phosphorylates the S61 and S94 sites of YAP. Phosphorylation at S94 inhibits the binding of YAP to TEAD, and the expression of downstream target genes of YAP is inhibited 53; the other is that AMPK promotes the expression of LATS2 through phosphorylation of angiomotin like 1 (AMOTL1) and activates the Hippo signaling pathway to cause YAP phosphorylation and inactivation 54.

Hypoxia is a stress condition closely related to pathological processes such as tumor development and myocardial injury. Under hypoxic conditions, the E3 ubiquitin ligase SIAH2 could bind to LATS2 and undergo ubiquitination modification. The expression of LATS2 is degraded, thereby activating YAP/TAZ into the nucleus and increasing the secretion of PGE2 (prostaglandin 2) 55. YAP is down-regulated in neuroma blasts after brain hypoxia-reoxygenation (HR) injury. Overexpression of YAP could block the apoptotic signal associated with mitochondria and inhibit HR-mediated neuroma cell death 56. The upstream kinase MST1 plays an important role in reactive oxygen, which could induce cell death, reactive oxygen defense, and could be activated under oxidative stress conditions 2,25,26. Under pro-oxidant conditions, activating transcription factor 4 (ATF4) inhibits the expression of E3 ubiquitin-protein ligase Nedd-4 (NEDD4.2) and WW domain containing E3 ubiquitin protein ligase 1 (WWP1) in mRNA levels, and the LATS1 proteasome-mediated by encoding ubiquitin ligase would be degraded 57. As a transcriptional coactivator of forkhead box O1 (FOXO1), YAP could directly bind to FOXO1 and activate FOXO1-mediated transcription of catalase and superoxide dismutase 2 (SOD), reducing heart damage by oxidative stress and ischemia-reperfusion 58.

### Regulation of Hippo signaling pathway by virus

A virus is a non-cellular life form with a simple structure containing only one nucleic acid (DNA or RNA), which replicates, transcribes, and translates in the host cell. When it enters the host cell, self-replication begins and affects the gene expression and energy metabolism of the host cell. Hippo signaling pathway also plays a critical negative regulatory role in virus-induced diseases. Many human viruses exert their carcinogenic functions by activating the activities of YAP and TAZ. One study found that Human papilloma virus (HPV) virus induces degradation of Scribble protein by HPV E6 oncoprotein, which leads to the development of cervical cancer 59. In addition, YAP is more likely to localize to the nucleus in HPV-positive oropharyngeal squamous cell carcinoma than in precancerous tissues, which is also associated with the development of squamous cell carcinoma of the oropharynx 60. HBV recognized by Toll-like receptors can activate NF-κB and Hippo signaling in both murine and human hepatocytes, thereby inducing innate immune mechanisms 61. In addition, EBV-infected gastric cancer cells can be observed to have greatly increased YAP activity and elevated expression of downstream target genes of the Hippo signaling pathway after 12-24 hours. Meanwhile, in breast cancer, EBV infection also causes inactivation of the Hippo signaling pathway 62. It also has been reported that when hepatitis C virus (HCV), infectious mollusc virus (MCV), mouse polyomavirus (MupyV), Kaposi Sarcoma-associated herpesvirus (KSHV), Zika virus (ZIKA) affecting host cells, the increase of YAP/TAZ expression activity could be found 63.

## The downstream regulatory network of the Hippo signaling pathway

**Figure 3:**
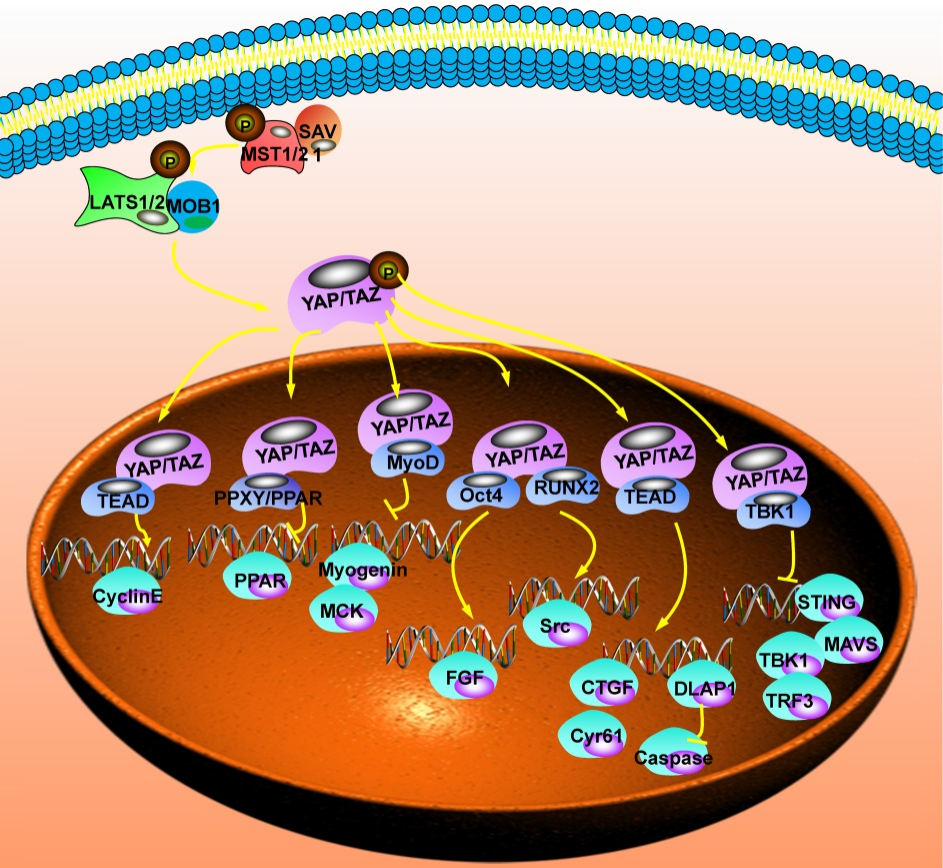
** The downstream regulatory network of the Hippo signaling pathway.** After YAP enters the nucleus, it needs to be combined with other proteins to form a complex before it could regulate transcription. After that, it could inhibit the transcription of peroxisome proliferator activated receptor (PPAR), creatine kinase (MCK), non-receptor tyrosine kinase (Src) and stimulator of interferon response cGAMP interactor 1 (STING). It could also promote the transcription of Cyclin E, epidermal growth factor (EGF), Connective tissue growth factor (CTGF), cellular communication network factor 1 (CCN1) and Drosophila melanogaster (DLAP1).

YAP/TAZ, the Hippo signaling pathway's main effector, does not contain DNA binding sites and is a cofactor for transcription activation. Combining with TEADs, p73, erb-b2 receptor tyrosine kinase 4 (ERBB4), early growth response 1 (EGR-1), T-box transcription factor 5 (TBX5), SMADs, and other transcription factors could activate the transcription of downstream target genes. Therefore, its transcriptional association domain determines the biological function of YAP/TAZ and the selection of its target genes. The functions of interacting proteins are different, and the downstream mechanisms of YAP/TAZ involved in regulation are also different 64 (Fig. 3).

### Regulation of Hippo signaling pathway on cell growth

Connective tissue growth factor (CTGF) is the direct target gene of YAP-TEAD. There are 3 TEAD binding sites in its promoter sequence, making TEAD directly bind to the CTGF promoter 65. In breast cancer cells, the anti-apoptotic proteins Bcl-xL, cIAPI, and survivin are activated by CCN1 and CTGF, which could antagonize taxane-induced apoptosis. Similarly, the promoter activity of YAP/TAZ is activated by CCN1 and CTGF, which could antagonize the function of taxane 66. The PDZ binding motif plays an important role in the biological functions of transcription coactivators. The loss of the PDZ binding motif could considerably prevent the oncogenic transformation induced by YAP (5SA) and the nuclear localization of CTGF expression. This effect is mediated by TEAD-mediated CTGF transcription 67.

Tissue fibrosis is a pathological status that is connected to the impaired epithelial repair and excessive deposition of extracellular matrix (ECM). In fibroblasts, ECM stiffness could mechanically activate the expression of YAP/TAZ, thereby promoting the production of fibrotic mediators and fibroblast growth factor (FGF), leading to tissue stiffness, and establishing a feedforward loop for fibroblast activation and tissue fibrosis 68. VEGF plays a crucial function in organ development and early tumor angiogenesis, promoting its growth and spread. YAP/TAZ mediates the signal transduction of VEGF. Simultaneously, the VEGF signal affects GTPase activity, which contributes to the activation of YAP/TAZ. This positive feedback control leads the changes in transcription and cytoskeleton, and finally promotes tumor cell angiogenesis 68.

### Regulation of Hippo signaling pathway on cell differentiation

The differentiation of myoblasts is essential for forming skeletal muscle and is regulated by a series of transcription factors (including myogenic differentiation factor 1 (MyoD), Myogenin and transcription co-regulators). Through the interaction between the WW and MyoD, TAZ (transcriptional coactivator with PDZ binding motif) could strengthen the binding of MyoD and myogenin gene promoter, which could activate and promote the transcription of myogenin and MCK genes. Finally, the terminal differentiation of muscle cells is induced. The MEK5/ERK5-MAPK cascade is essential for muscle cell differentiation. Muscle differentiation could promote by YAP by activating the Abl/Src/MEKK3/MEK5/ERK5 kinase cascade 69. The high activity of YAP in muscle fibers will not cause fiber hypertrophy or fiber type changes but will cause reversible atrophy and degeneration 70. As a necessary transcription factor, RUNX2 could stimulate bone formation and promote bone cell differentiation in combination with TAZ. The YAP molecular structure has an SH3 binding domain, which TAZ does not have. It could make YAP1 directly bind to Src. When the activity of Src is inhibited, it could induce osteoblast differentiation.

There are currently two research results. One is that YAP1 acts as a transcriptional co-repressor of RUNX2 on the osteocalcin promoter in response to Src stimulation, which mediates the phosphorylation of YAP tyrosine sites. And this could promote the binding of YAP and RUNX2. The other is that YAP located in the nucleus, acts as a transcriptional coactivator of RUNX2, and promotes bone formation 71. PPARγ is a ligand-activated receptor in the nuclear hormone receptor family. PPARγ is a ligand-activated receptor in the nuclear hormone receptor family. As an important cell differentiation transcription factor, PPARγ has two molecular mechanisms involved in regulating the Hippo signaling pathway. One is that TAZ directly binds to the PPXY motif of PPARγ to inhibit the active expression of PPARγ. The other is the upstream kinase SAV1 competing with TAZ to antagonize the same PPXY motif that binds to PARγ. At the same time, SAV1 promotes the phosphorylation of TAZ through the Hippo pathway. The retention of TAZ in the cytoplasm inhibits the nuclear binding of TAZ and PPARγ 72. Notch signal is a critical factor in epidermal differentiation, triggering proliferation, or cell differentiation in different cell types. Cell morphology and extracellular matrix could regulate the activity of YAP/TAZ, and mechanical activation of YAP/TAZ could inhibit the differentiation of epidermal stem cells by inhibiting Notch signaling 73.

### Regulation of Hippo signaling pathway on apoptosis

The Hippo signaling pathway regulates the drosophila apoptosis inhibitor (DIAP1), which mediates caspase's inactivation to inhibit cell apoptosis 58. At the same time, microRNA Bantam in Drosophila is also a downstream target gene of the Hippo signaling pathway, regulating growth and tumorigenesis by inhibiting the translation of the apoptotic factor- Hid 74. Also, YAP1 regulates anaplastic lymphoma kinase (ALK) by inhibiting the anti-apoptotic factors MCL1 apoptosis regulator and Bcl-xL (B cell leukemia/lymphoma), which has clinical significance for molecular targeted therapy in non-small cell lung cancer with ALK-positive mutations. In terms of the cycle, the Hippo pathway is highly inactivated in glomerular mesangial cells cultured in high glucose *in vitro*. The reduction of MST1/2 and LATS1/2 phosphorylation promotes YAP expression and nuclear entry. It increases the YAP-TEAD interaction and activates the expression of downstream target genes such as cyclin E 75. In embryonic neuroblasts, the Ama-Nrt-Abl pathway could also regulate cyclin E expression by changing the function of the Hippo pathway effector YAP 75.

### Regulation of Hippo signaling pathway on cellular immune factors

Hippo signaling pathway plays an integral part in the immune system and a variety of functional immune cells, especially in the process of immune cells responding to the virus, bacterial invasion, or tumorigenesis and maintaining their homeostasis. In terms of antiviral defense, YAP/TAZ could form a complex with TANK-bindingkinase1 (TBK1) and effectively prevent the K63 ubiquitination modification of TBK1. Moreover, it also could form STING/MAVS-TBK1-IRF3 complex 76. In terms of immunity, YAP/TAZ often does not require the regulation of upstream kinases and directly affects T cells. At the same time, it could regulate the crosstalk between immune cells and tumor cells in the tumor microenvironment by inhibiting the polarization of bone marrow cells and macrophages. Besides, the important immunosuppressive factor PD-1 could bind to the upstream kinase MOB1 of the Hippo pathway and inhibit MOB1 phosphorylation. The downstream targets of the Hippo pathway, CYR61, and CTGF, are directly involved in immune regulation mediated by PD-1 76.

## Tumor phenotypes affected by the Hippo signaling pathway

When external signals activate the Hippo signaling pathway, it may inhibit cell proliferation and promote cell apoptosis, which could also control the size of tissues and organs and inhibit tumor formation. When the Hippo signaling pathway is abnormal, it will participate in the signal transduction of Wnt, Notch, EGFR, TGF-β, JAK-STAT, and other pathways. Thereby, it could affect the occurrence and the development of various human tumors, such as lung cancer, breast cancer, gastric cancer, hepatocellular carcinoma, renal cell carcinoma, colorectal cancer, etc.

### Hippo signaling pathway promotes cell proliferation

Cell proliferation is an essential feature of organisms. Cells divide to produce new cells to supplement aging or dead cells in the body. The strict control of cell division is essential to maintain the normal development of cells and the homeostasis of the cell environment. Abnormal cell proliferation is related to a variety of pathological conditions. The Hippo signaling pathway plays a significant part in regulating cell proliferation without relying on organ size control. Studies on Drosophila/mouse have found that the inactivation of tumor suppressor genes in the Hippo signaling pathway or the activation of the oncogene Yki/YAP will lead to excessive tissue growth. The characteristic performance is increased cell differentiation and inhibition of cell apoptosis.

The immunohistochemical method could be used to detect the expression of YAP in non-small cell lung cancer cases. The results showed that YAP was highly expressed in the nucleus in adenocarcinoma, and it was positively correlated with the expression of cyclin A and mitogen-activated protein kinase. The pathological TNM staging study of squamous cell carcinoma found that the expression of YAP in the cytoplasm of stage I was higher than that of stages II to IV. It suggests that the high expression of YAP in the nucleus is closely related to the cell cycle, promoting the proliferation of renal cancer cells. This indicated that the high expression of YAP in the cytoplasm could be used as an independent predictor of clinical pathological staging. After overexpression of MST1 in lung adenocarcinoma A549 cell line, cell growth was inhibited, and the anti-proliferation ability was related to YAP phosphorylation. After the YAP gene was explicitly knocked out, the proliferation of hepatocytes was inhibited, and even necrosis occurred. Unusual concentrations of bile acids could perform the role of upstream activators of the Hippo signaling pathway, promote the expression of scaffold protein IQGAP1, and activate YAP transcriptional activity to stimulate liver growth and tumorigenesis 77.

### Hippo signaling pathway inhibits cell apoptosis

Apoptosis, also known as programmed cell death (PCD), refers to the autonomous and orderly death of cells under pathological and physiological conditions, in order to maintain a stable biological environment 78. In hepatocellular carcinoma and intrahepatic cholangiocarcinoma cells, YAP and survivin are highly expressed in nuclei. Moreover, survivin is an inhibitor of apoptosis protein, and the expression of survivinm RNA depends on YAP protein 79. After overexpression of MST1 *in vitro*, YAP phosphorylation increased, and mRNA expression of ETGF, amphiregulin (AREG), and survivin was all down-regulated. After that, cell proliferation was inhibited, and apoptosis was induced 80. After knocking down the TAZ gene *in vitro*, the expression of cyclin A and CTGF decreased significantly, the cell cycle was arrested in the G0~G1 phase, and cell proliferation was inhibited. At the same time, it was accompanied through a considerable rise in the expression of caspase3 and induced apoptosis, showing the ability of TAZ to promote cell proliferation and inhibit cell apoptosis 81,82.

### Hippo signaling pathway promotes cell invasion and metastasis

Cell migration is the basis for many important physiological activities of cells and an important step and key link in pathological processes such as inflammation and tumorigenesis 83,84. Enhanced YAP/TAZ activity could promote tumor cell migration 85. As a phospholipid messenger between cells, lysophosphatidic acid (LPA) could activate G protein-coupled receptors and take part in the normal physiological functions of the body. In ovarian cancer, the Hippo signaling pathway is complicated in LPA-mediated migration of ovarian cancer cells, manifested by LPA-induced dephosphorylation of YAP into the nucleus to function. This response relies on the participation of multiple regulatory factors such as Rho/Rock, PP1A, AREG, and EGF. Using siRNA knockdown YAP, YAP expression activity decreases, and LPA-induced ovarian cancer cell metastasis and invasion ability are also significantly reduced 82.

TEAD is the downstream effector YAP/TAZ of the Hippo signaling pathway. The interacting protein is essential for YAP/TAZ-mediated tumor growth and metastasis. The invasion ability of metastatic breast cancer is closely related to TEAD transcription activity. In non-metastatic tumor cells, enhancing the transcriptional activity of YAP-TEAD could lead to non-metastatic NMuMG cells, 67NR cells, and A375 cells to metastasize to the primary site 86. In breast cancer cells, overexpression of TAZ can regulate cell proliferation, migration, and epithelial-mesenchymal transition. The interaction of TAZ-TEAD enhances the activity of the bone morphogen-netic protein 4 (BMP4) promoter, and the activated BMP4 also, in turn, increases the expression activity of TAZ. Western blot and wound healing analysis showed that TAZ overexpression could induce cell migration and increase. pSmad1/5 expression activity while knocking out the target gene BMP4 gene significantly reduces cell migration ability 87,88.

## Possible tumor suppressor targets in the Hippo signaling pathway

**Figure 4:**
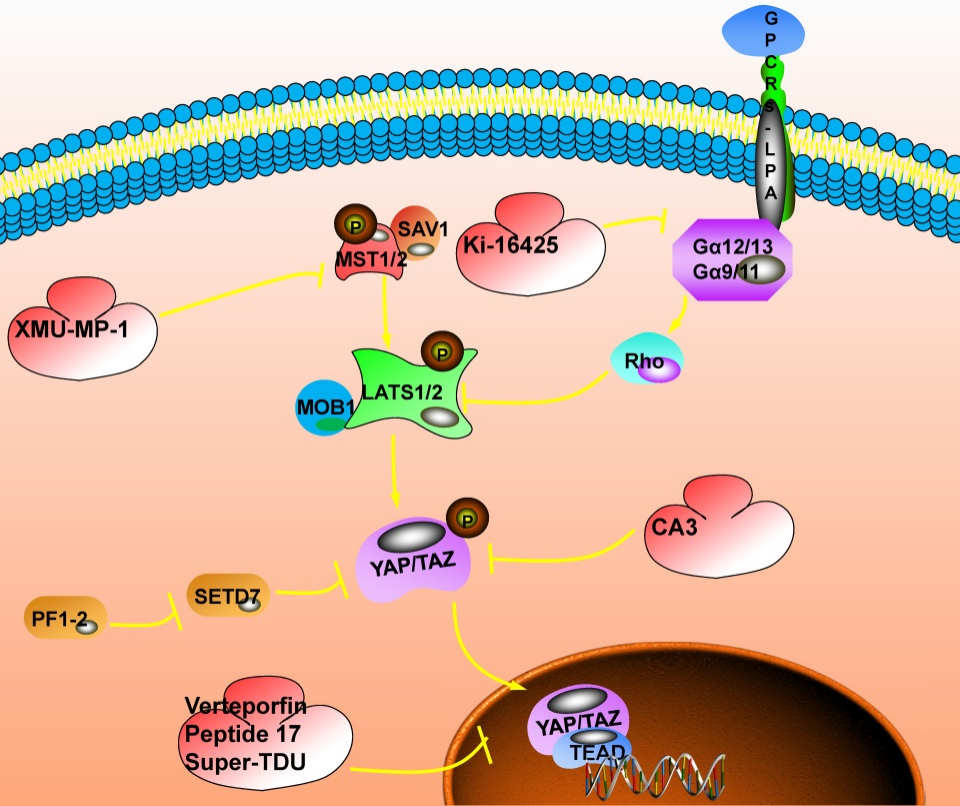
** The inhibitory target involved in the Hippo signaling pathway.** The switch that closes the Hippo pathway is whether YAP enters the nucleus. When YAP is phosphorylated, it will be stagnated in the nucleus and the Hippo pathway will be activated; when YAP enters the nucleus, the Hippo pathway will be closed, which could promote the occurrence of tumors. The current targeted inhibitors have two ideas, one is to promote the phosphorylation of YAP. For example: XMU-MP-1 could inhibit the phosphorylation of MST1/2, promote the cascade of MST1/2, LAST1/2, and YAP, and promote the phosphorylation of YAP; Ki-16425 could inhibit the phosphorylation of LAST1/2 and promote the phosphorylation of YAP; CAS could directly promote phosphorylation of YAP. Another idea is to inhibit the interaction of YAP with TEAD after entering the nucleus. Here, Verteporfin compounds could be used to achieve this function.

The subcellular localization of YAP/TAZ is a key determinant of its transcriptional regulation and signal transduction, which provides an attractive target for cancer therapy. YAP/TAZ is often over-activated due to the disorder of the Hippo signaling pathway. It has been confirmed that the activation or overexpression of YAP/TAZ is related to the poor prognosis of cancer patients. The inhibition of YAP/TAZ could improve the prognosis of cancer patients. Therefore, the development of YAP/TAZ inhibitors based on the Hippo signaling pathway is expected to become a cancer treatment target (Fig. 4).

### Targeting inhibition of upstream kinase molecules in the Hippo signaling pathway

It is difficult to use small molecule target the unstructured nature of YAP and TAZ. Therefore, YAP/TAZ inhibitors tend to target the upstream or downstream molecules of YAP. Here we divide inhibitors into two categories, one is upstream-targeting inhibitors, and the other is downstream-targeting inhibitors. Here we focus on targeting upstream inhibitors, and the next section on targeting downstream.

MST1/2 is an essential cascade kinase in the Hippo signaling pathway, and XMU-MP-1 is a highly effective and selective inhibitor of MST1/2 (MST1, IC50=71.1nM; MST2, IC50=38.1nM). *In vitro* studies have shown that in mouse models of acute or chronic liver injury, the addition of XMU-MP-1 can effectively activate the protein expression of the downstream effector YAP, thereby enhancing intestinal repair in mice and promoting liver repair and regeneration^89^. In the study of head and neck squamous carcinoma, it was found that FAT1 is directly related to MST1, the assembly of the Hippo kinase core complex, which leads to the subsequent phosphorylation of LATS1/2 and YAP^89^.At the same time, PIK3CA could activate YAP/TAZ through the PIK3-PDK1 axis to mediate different stimuli (EGFR, FAK, fibronectin, GPCRs, etc.) and inhibit some BET protein inhibitors such as Birabresib, which inhibit the bromine domain and extra terminal domain of the protein. This is expected to become a new target for blocking the Hippo signaling pathway to treat cancer.

In addition, some molecules that target surface receptors could also inhibit the activation of the Hippo signaling pathway. Such as EGFR-targeting inhibitors, erlotinib 90,91 and AG-1478 92; G-protein-targeting coupled receptors, losartan and dihydrohexidine; targeting of antibody-BHA 2.1 93 and clone AIIB2 94, RGD peptide 95 and cyclic RGD peptide 96; Inhibitors targeting VEGFR, such as SU4312, apatinib, axitinib and pazopanib, and the forskolin. Some actin-targeted inhibitors that can promote YAP nuclear localization can also be used to block YAP from entering the nucleus, such as latrunculin A 97, cytochalasin D 98,99, blebbistatin and ML-7. In the same way, okadaic acid or calmodulin A can over-express the phosphatase that helps phosphorylate YAP, thereby trapping more YAP in the cytoplasm.

Some metabolism-related molecules have also been used as targets to block the Hippo signaling pathway. The activity of YAP can be inhibited by targeting focal adhesion kinase (FAK), Src and integrin-linked kinase (ILK). In addition, AMPK activator A769662 and AICAR (an AMP mimic) are also inhibitors of YAP 100-102.

### Targeting the inhibition of downstream transcription factors in the Hippo signaling pathway

The downstream effector YAP of the Hippo pathway requires the coordination of transcription factors to regulate cell proliferation, anchorage-independent growth, and epithelial-mesenchymal transition. Among them, TEAD is an important binding partner of YAP to exert its carcinogenic activity. Inhibition of YAP/TEAD activity *in vivo* could effectively inhibit the biological functions caused by the YAP-TEAD interaction. The introduction of cysteine at positions 87 and 96 of YAP could induce disulfide formation, and the change of the peptide significantly improves the ability to disrupt the YAP-TEAD interaction *in vitro*. Similarly, introducing a dominant-negative mutation (Y406H) of TEAD could also disrupt the YAP-TEAD interaction. At this time, the tumor growth rate in the hepatocellular carcinoma xenograft model was significantly reduced, confirming that it is an effective method to prevent the formation of the YAP-TEAD complex by directly targeting TEAD 103.

In addition, *in vitro* selection of appropriate inhibitors for YAP-TEAD interaction is also a useful breakthrough point 104,105. The small molecule compound Verteporfin (VP) could inhibit the YAP-TEAD interaction and show a significant inhibitory effect on TEAD transcription activity. At the same time, the expression level of YAP/TAZ is also significantly reduced 106. VP arrests the cell cycle in the G1 phase and induces apoptosis in a dose- and time-dependent manner. Mechanism studies have shown that VP damages the YAP-TEAD interaction, thereby inhibiting the expression of target genes and inhibiting cell proliferation 107. Peptide17 also showed a strong affinity with TEAD *in vitro* experiments, effectively destroying the interaction of YAP-TEAD, and has its potential application value in YAP-related cancers 108. An FDA-approved verteporfin drug could also inhibit the YAP-TEAD interaction and inhibit YAP-induced overgrowth of cancer cells *in vivo* and *in vitro*.

The TDU domain of vestigial like family member 4 (VGLL4) is a natural antagonist of YAP, which competes with YAP to bind TEAD 109. Super-TDU could specifically target YAP/VGLL4 tumor cells with a high ratio, reduce the endogenous YAP-TEAD interaction, and down-regulate the expression of target genes such as CTGF, CCN1, and caudal type homeobox 2 (CDX2) 110,111. Carbonic anhydrase 3 (CA3) prevents the transcriptional activity of YAP-TEAD explicitly. In esophageal adenocarcinoma cells, the combination of CA3 and 5-FU could synergistically inhibit YAP expression and the growth of esophageal adenocarcinoma cells. It indicates that CA3 is a new inhibitor of YAP 111,112. In addition, drugs targeting downstream YAP/TAZ targets (BCL-xL, FOXM1 and TG2) could also be used to combat YAP/TAZ-mediated carcinogenesis, such as A37 113, celecoxib 114, TP-0903 115, cyclic peptide RA-V (deoxybouvardin) 116, navitoclax 117, thiostrepton 118 and NC-9 119,120.

### Molecular inhibition of targeting Hippo signaling pathway

The occurrence of cancer will show changes in various signaling pathways, and these signaling pathways have modes of co-occurrence or mutual exclusivity. 89% of tumors have at least one driving change in these pathways, which reminds new ideas for combination therapy in cancer. The methyltransferase SET domain containing 7 (SETD7) plays a central regulatory function in the Hippo/YAP pathway and Wnt/β-catenin pathway to control tumors' occurrence. Similarly, YAP is also involved in the activation of the Wnt pathway. PFI-2 is a highly effective and selective inhibitor of SETD7 methyltransferase. In (R)-PFI-2 pretreated human breast cancer cell MCF7, YAP's location changed rapidly. It reveals the effect of the lack of phenotypic SETD7 on the Hippo signaling pathway, indicating that the methyltransferase activity of SETD7 continuously and dynamically regulates YAP 112.

Lysophosphatidic acid (LPA) is a biologically active lysophospholipid and participates in a variety of physiological reactions. In studying LPA receptors' functional expression in salivary gland epithelial cells, it was suggested that LPA receptors could activate the expression of downstream effectors YAP/TAZ through LATS/MOB1 and RhoA/ROCK. Up-regulation of the expression activity of the downstream genes CTGF, NKRD1, and CCN1 targeted by YAP/TAZ were observed in LPA-treated cells 121. In the study of LPA-induced cell contraction involved in the extracellular matrix (ECM), it was found that LPA and its receptor promote the YAP/TAZ transcriptional activity *in vitro* by regulating cell contraction tension and enhance the expression of CTGF, which in turn leads to ECM increase 122. Ki-16425 is a competitive and effective LPA reversible inhibitor. In the cell line, it could block LPA receptors to induce the dephosphorylation of YAP/TAZ and inhibit the Hippo pathway. However, the Hippo signaling pathway is critical to physiological homeostasis and regeneration after tissue injury. Tumor treatment drugs for Hippo pathway inhibition may cause potential damage to patients with co-morbidities. Therefore, caution should be taken in the selection and dosage of Hippo pathway inhibitors 123,124. Unlike bulk materials, nanoparticles exhibit unique properties such as large specific surface area, electromagnetic properties, and optical behavior. These characteristics enable them to solve many problems faced by traditional diagnosis and treatment methods, which are mainly manifested in the following aspects: First, during the preparation process of nanoparticles, the composition, size, shape, and surface properties could be adjusted artificially, so that nanostructures with specific properties could be synthesized as needed to encapsulate and protect anti-cancer drugs. Secondly, by controlling the shape, size, and surface modification of nanoparticles, the drug-loaded nanoparticles could be targeted to reach specific lesions. They could release drugs in a controlled manner without affecting normal tissues and organs. This solves the problem of traditional therapies, including the systemic toxicity and low treatment efficiency. Finally, some nanoparticles have excellent magnetic or optical properties. They could be used for biological imaging (such as magnetic resonance imaging or fluorescence imaging, etc.) to integrate diagnosis and treatment in the body. However, as of now, no nano-drug targeting the Hippo pathway has been developed. It could be hoped that future Hippo pathway research could also focus on nano-strategies. However, so far, no nano-drug targeting the Hippo pathway has been developed, and it is hoped that future Hippo pathway research could focus on nano-strategies.

## The other biological effects of the Hippo signaling pathway

In addition to tumor-related regulation, the Hippo pathway is also closely related to many other biological processes. In recent years, in the Hippo signaling pathway's research directions, the focus of research has shifted from the initial screening of core regulatory factors to functional biological research, which is mainly reflected in organ development, tissue regeneration, stem cell self-renewal, tumor occurrence and development.

### The Hippo signaling pathway involved in regulating organ size

Hippo signaling pathway participates in and mediates the development of human organs and carcinogenesis 112,125. For example, after specific overexpression of YAP in transgenic mice's liver, abnormal liver enlargement was observed. After the overexpression of YAP was terminated, the liver's specific gravity slowly returned to a normal level. Similarly, by specifically knocking out the upstream kinase MST1/2, SAV1, or neurofibromin 2 (NF2) in the Hippo pathway, abnormal liver enlargement was observed. However, changes in the Hippo signaling pathway will not cause changes in all organs. For example, after specifically knocking out MST1/2 in mice, except for abnormal hyperplasia of liver, heart, stomach, and spleen, there is no apparent hyperplasia of kidney, lung, and limbs. While specifically knocking out YAP expression in the intestine, no obvious defects were found in the intestinal tissue structure and size. Besides, the loss of YAP function has no effect on breast development during adolescence, but it will seriously hinder the growth and development of the breast during pregnancy. These studies suggest that the Hippo signaling pathway is involved in regulating organ size and depends on the type of organ.

### The Hippo signaling pathway involved in regulating tissue regeneration

Under physiological and pathological conditions, the key to wound healing is the generation and maintenance of blood vessels. YAP/TAZ participates in the formation of endothelial cells, vascular barriers, and vascular remodeling. The regulation of angiogenesis plays a central role in the restoration of the body after the treatment of various diseases. Therefore, although the normal growth and development of tissues and the maintenance of homeostasis do not depend on the active expression of YAP/TAZ, tissue regeneration after tissue injury needs to depend on the active expression of YAP/TAZ 126,127. For example, nude mice injected with dextran sodium sulfate-induced colitis could effectively regenerate and repair after a period of feeding. While YAP expression in nude mice is specifically knocked out, it cannot effectively regenerate and repair after a period of feeding. Similarly, after partial excision of 70% of the mouse liver, the liver with strong regenerative capacity can also use the remaining part to regenerate and recover. During this period, YAP's expression level and activity increased significantly and remained at a high level for a long time. MST1/2 and LATS1/2 expression activity decreased. When the liver regeneration is restored, the activities of YAP, MST1/2, and LATS1/2 return to the level of the resting state of the liver, which indicates that the Hippo signaling pathway is significant for maintaining a typical liver weight ratio and liver homeostasis. Compared with intestinal tissue and liver, adult and adult mouse heart tissue's regenerative ability is minimal. But inhibition or inactivation of the Hippo signaling pathway and overexpression of YAP could improve cardiomyocytes' regenerative ability to a certain extent. Conversely, the specific deletion of YAP will significantly impair the regeneration ability of the neonatal mouse heart.

In short, the Hippo signaling pathway has a regulatory effect on the regeneration process of various tissues. In addition, since cell biology and biomaterial technology are the basis of cell tissue regeneration therapy, biomaterials may play a vital role in affecting cell recoding and fate by triggering gene expression. Understanding the interaction between cells and biomaterials may provide a theoretical basis for biomaterials designed for clinical applications in tissue regeneration.

### The Hippo signaling pathway involved in regulating the self-renewal of stem cells

Cancer stem cells have the potential for self-renewal and transformation, which is the main reason for patients' poor prognosis. The Hippo signaling pathway is involved in regulating normal organ development and progenitor cell differentiation 128. YAP/TAZ is highly expressed in stem cells of various tissues, suggesting that YAP/TAZ is involved in stem cell self-renewal and conversion. Overexpression of YAP or the inactivation of upstream inhibitors of the Hippo pathway activates the expression of YAP. It is found that the progenitor cell expansion and differentiation ability are weakened, and the skin, intestine, and other tissues are abnormally proliferated. Five days after YAP overexpression, the differentiation ability of intestinal stem cells was weakened, and the overexpression of YAP was stopped simultaneously. It was found that the differentiation ability of intestinal stem cells was restored.

Similarly, the phenotype of MST1/2 knockout in mice is the same as that of YAP overexpression, which indicates that YAP is participated the regulation of intestinal stem cell self-renewal and differentiation. In the early development of the skin, YAP is distributed in the nucleus of a single layer of skin cells as the skin begins to stratify. YAP translocates to basal cells with strong proliferation ability, and the expression activity decreases with cell proliferation. When YAP is overexpressed in skin progenitor cells, the skin tissue layer becomes thicker, and basal cells increase. At the same time, the cells that could proliferate are no longer limited to the basal layer. In addition, the ability of progenitor cell clone formation is enhanced, suggesting that stem cells have enhanced self-renewal. When specifically knocking out YAP or interfering with the expression of TEAD, the skin tissue layer becomes thinner, and the formation of multiple skins such as limbs, eyes, mouth, and nose is hindered, and basal cells are reduced. These results suggest that the Hippo signaling pathway could promote the self-renewal of epithelial stem cells and inhibit terminal differentiation.

## Conclusions

The Hippo signaling pathway regulates organ development, tissue regeneration, and stem cell self-renewal. It does not act solely but interacts with other signaling pathways. The Hippo signaling pathway is also complicated in the occurrence and development of various human cancers. The nuclear localization and overexpression of YAP/TAZ have been confirmed in many human cancers. However, the underlying mechanism of Hippo pathway dysregulation and YAP/TAZ activation is still not well understood. Much efforts are needed to continue to study the biological functions of the Hippo signaling pathway in-depth, which is beneficial to the future research and treatment of tissue regeneration, stem cell diseases, and cancers. Through regulating it, the specificity and targeting of tumor treatment will be significantly improved, and at the same time, the damage of normal stem cells by drug treatment is expected to be significantly decreased.

## Abbreviations

MST1/2: mammalianSTE20-like protein kinase
SAV1: human Salvadorhomology 1
LATS1/2: large tumor suppressor1/2
MOB1: MOB kinase activator
YAP: Yes-associated protein
TAZ: Tafazzin
TEADs: TEA domain family members
NDR1/2: Late embryogenesis abundant (LEA) hydroxyproline-rich glycoprotein family
FOXO1: forkhead box O1
TJs: tight junctions (TJs)
AJs: adhesive connections
DUBs: ubiquitin-specific protease family
USP9X: ubiquitin specific peptidase 9 X-linked
AMOT: angiomotin
FAT: FAT atypical cadherin 1
Ex: Expande
PCP: Planar Cell Polarity
GPCRs: G Protein-Coupled Receptors
PARs: G protein-coupled receptor-protease activated receptor
IGF-1: insulin like growth factor 1
PI3K: phosphatidylinositol 3-kinase
VEGF: vascular endothelial growth factor
AMPK: Protein kinase AMP-activated catalytic subunit alpha 1
AMP: adenine phosphoribosyltransferase
AMOTL1: angiomotin like 1
PGE2: prostaglandin 2
HR: hypoxia-reoxygenation
ATF4: activating transcription factor 4
NEDD4.2: E3 ubiquitin-protein ligase Nedd-4
WWP1: WW domain containing E3 ubiquitin protein ligase 1
FOXO1: forkhead box O1
SOD: superoxide dismutase 2
HBV: hepatitis B virus
HCV: hepatitis C virus
MCV: infectious mollusc virus
HPV: human papillomavirus
Mupy V: mouse polyomavirus
KSHV: Kaposi Sarcoma-associated herpesvirus
EBV: Barr virus
ZIKA: Zika virus
ERBB4: erb-b2 receptor tyrosine kinase 4
EGR-1: early growth response 1
TBX5: T-box transcription factor 5
EGF: epidermal growth factor
FGF: fibroblast growth factor
CTGF: Connective tissue growth factor
CCN1: cellular communication network factor 1
DLAP1: Drosophila melanogaster
PPAR: peroxisome proliferator activated receptor
MCK: creatine kinase
Src: non-receptor tyrosine kinase
STING: stimulator of interferon response cGAMP interactor 1
ECM: extracellular matrix
MyoD: myogenic differentiation factor 1
DIAP1: drosophila apoptosis inhibitor
ALK: anaplastic lymphoma kinase
BCL2: B cell leukemia/lymphoma 2
TBK1: TANK-bindingkinase1
PCD: programmed cell death
AREG: amphiregulin
BMP4: bone morphogen-netic protein 4
FAK: focal adhesion kinase
ILK: integrin-linked kinase
AICAR: an AMP mimic
VP: Verteporfin
VGLL4: vestigial like family member 4
CDX2: caudal type homeobox 2
CA3: carbonic anhydrase 3
SETD7: SET domain containing 7
LPA: Lysophosphatidic acid
NF2: neurofibromin 2
